# Tomato FW2.2/CNR might regulate fruit size via plasmodesmata callose deposition

**DOI:** 10.1093/plphys/kiae251

**Published:** 2024-05-01

**Authors:** Thu M Tran, Kumari Billakurthi

**Affiliations:** Assistant Features Editor, Plant Physiology, American Society of Plant Biologists; Cold Spring Harbor Laboratory, Cold Spring Harbor, NY 11724, USA; Assistant Features Editor, Plant Physiology, American Society of Plant Biologists; Department of Plant Sciences, University of Cambridge, Cambridge CB2 3EA, UK

How do we grow a bigger fruit? This question excites everyone from backyard gardeners growing giant pumpkins for local fall festival contests to large-scale agriculture companies producing commercial fruit crops. Fruits are essential for plant reproduction, as they protect the developing seeds and aid in the dispersal of mature seeds. In agriculture, fruits are harvested and consumed. Despite the functional and agricultural importance of fruit size, its underlying genes and mechanisms still need to be better understood.

Tomato (*Solanum lycopersicum*) presents a wide variety of fruit sizes and shapes, which contributes to its popularity as one of the most highly consumed vegetables in the world. In addition to their fruit phenotypic diversity, tomatoes have extensive genetic resources and efficient genome editing, making them ideal systems for studying the mechanism of fruit size (Alonge et al. 2020). Thirty years ago, Frary et al. first isolated the gene underlying *FW2.2*, a major Quantitative Trait Loci (QTL) that accounts for up to 30% of tomato fruit size (Frary et al. 2000). *FW2.2* encodes a CELL NUMBER REGULATOR (CNR) family protein. CNR family proteins harbor an ancient eukaryotic placenta-specific (PLAC8) domain, and they exhibit diverse functions, including heavy metal transport, calcium uptake and signaling, and regulating organ size (reviewed in Beauchet et al. 2021). Their roles in regulating organ size, particularly in tomatoes, maize, and rice, are well established (Cong et al. 2002; Guo et al. 2010; Ruan et al. 2020). These previous studies suggest that CNR proteins act as negative regulators of the cell cycle in controlling organ size, although their precise molecular mechanisms remain to be elucidated.

In this issue of *Plant Physiology*, Beauchet et al. (2024) investigated the cellular and molecular mechanism underlying the mode of action of *FW2.2*. Using transient expression in tobacco leaves and stable transformation in tomato, they demonstrated that FW2.2 is enriched at plasmodesmata (PD) with N and C termini facing the apoplast. PD are plant-specific structures: plasma membrane–lined cytoplasmic channels connecting adjacent cells. These structures are essential for transporting nutrients, metabolites, and signaling macromolecules from cell to cell. The localization of FW2.2 suggested its potential involvement in cell-to-cell transport through PD.

To examine the roles on FW2.2 in cell-to-cell transport, the authors applied Drop-ANd-See (DANS) assays (Cui et al. 2015). The DANS assay utilizes membrane permeable, nonfluorescent 5(6)-carboxyfluorescein diacetate (CFDA) dye, which is converted to fluorescent membrane-impermeable 5(6)-carboxyfluorescein (CF) upon cleavage by cellular esterases. This assay allows visualization and quantification of PD permeability in plant leaves via fluorescent microscopy. The authors observed an enhanced diffusion of fluorescent dye in leaf cells of overexpressing *FW2.2* lines, demonstrating the role of FW2.2 in controlling PD permeability.

PD permeability is controlled in part by the deposition and degradation of callose around the neck of PD. To validate if FW2.2 controls PD permeability through callose deposition, the authors examined the callose level in the leaves of the overexpression of FW2.2 and loss-of-function lines. The results revealed that overexpression of *FW2.2* reduces callose accumulation at PD, potentially enhancing cell-to-cell diffusion, while loss-of-function *fw2.2* mutations show no impact on callose levels. These findings suggest that FW2.2 plays a role in modulating PD callose deposition.

The authors established that FW2.2 controls accumulation of callose at PD in leaves. As FW2.2 is a major allele regulating tomato fruit size (Frary et al. 2000), the authors investigated the fruit development and the callose deposition at PD in the pericarp in genotypes with altered levels of FW2.2. In contrast to previous reports (Frary et al. 2000), either gain-of-function or loss-of-function of FW2.2 did not significantly change the fruit size. This might be due to gene redundancy with the CNR family (reviewed in Beauchet et al. 2021). However, the locule number was reduced by the overexpression of FW2.2, while the number was increased in loss-of-function lines. These results suggest that misexpression of FW2.2 might have impacted the termination of cell division in floral meristems. Moreover, FW2.2 negatively regulated the callose deposition at PD within the fruit pericarp.

However, identifying FW2.2’s function in callose deposition raises another question: How does FW2.2 regulate callose deposition if it does not have callose synthesis enzyme activity? Using an immunoprecipitation mass spectrometry proteomics approach, the authors found that FW2.2 is part of a protein complex containing various callose synthases (CalS) known to regulate callose homeostasis at PD. This interaction between FW2.2 and CalS suggests a mechanism for balancing callose synthesis at PD, with FW2.2 potentially negatively regulating CalS activity (Fig.).

**Figure.**
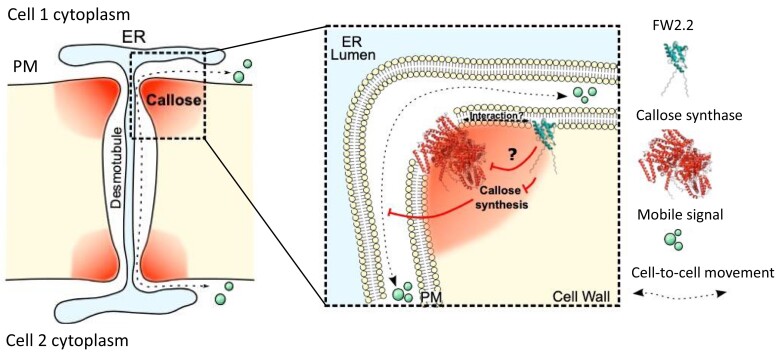
FW2.2 regulates callose deposition at PD via interactions with the callose synthase protein complex. FW2.2 negatively regulate callose synthase activity, thus impacting PD permeability and facilitating cell-to-cell symplastic transport. The figures are adapted from Beauchet et al. (2024).

In summary, this study discovered the role of FW2.2 in cell-to-cell communication via callose deposition at PD. It opens up some questions: how does FW2.2 regulation of PD permeability affect fruit size? Moreover, how is FW2.2 regulation of PD permeability associated with cell division? And which signaling molecules are diffusing through PD? The authors speculate, by modulating the PD permeability, FW2.2 might contribute to the diffusion of signaling molecules that play important role in determining the fruit size. Although it is not clear which cell cycle regulators pass through PD, it has been reported that Kip-Related Proteins that are major repressors of cell proliferation act non-cell-autonomously (Weinl et al., 2005). Furthermore, the study by Ruan et al. (2020) demonstrated the interaction of Kip-Related Proteins with FW2.2 in rice. Further studies are needed to determine whether FW2.2 regulates fruit size via symplastic transport of Kip-Related Proteins.
